# Microsurgical repair of severed thoracic spinal cord and clinical outcome: technical case report

**DOI:** 10.1186/s41016-022-00286-0

**Published:** 2022-07-25

**Authors:** Chandrasekaran Kaliaperumal

**Affiliations:** Department of Neurosurgery, The Royal Infirmary of Edinburgh, Little France, Edinburgh, EH16 4XA UK

**Keywords:** Spinal injury, Stab injury, Penetrating injury to spinal cord, Spinal cord repair ASIA score, Brown-Séquard syndrome

## Abstract

**Background:**

This report describes a case of successful repair of severed thoracic spine in a young man who presented with a penetrating stab injury to spine resulting in Brown-Séquard syndrome. Surgical technique and post-operative management is discussed.

**Case presentation:**

A 34-year-old fit and well healthy man was admitted with a history of stab injury to the thoracic spine at thoracic T2/3 level with ASIA impairment score (AIS) score D with an incomplete spinal cord affecting his left lower limb with complete paralysis and right lower limb paresis with impaired sensation below T6 level to L5. Neuroimaging confirmed a penetrating knife injury traversing the T2/3 level causing hemi-section of the spinal cord confirmed intraoperatively. He underwent an urgent exploratory surgery of his spine and a T2/3 laminectomy was performed to aid removal of the knife. The dura was noted to be contused and severed spinal cord was noted to be severed with associated cord oedema. A microsurgical repair of the severed cord was performed with duroplasty followed by intense neuro-rehabilitation. On a 3 month follow up his AIS score is E with lower limb power is 5/5 bilaterally and he is able to mobilise independently up to 8–10 steps without any supportive aid and with crutches he is independently functional and mobile.

**Conclusion:**

This is the first documented case of microsurgical repair of severed thoracic spinal cord secondary to traumatic knife injury. In the management of such scenario, apart from the removal of foreign body, repair of the cord with duroplasty should be carefully considered. The role of spinal neuroplasticity in healing following timely repair of the spinal cord along with intense rehabilitation remains the key. This had resulted in a good clinical and functional outcome with in a 18-month follow up.

## Background

Spinal cord injury (SCI) is a severe neural trauma and depending on the damaged segment and severity of the trauma, it is classified into complete and incomplete SCI. SCI is also a debilitating neurological condition with tremendous socioeconomic impact on the affected individuals and the health care system. Etiologically, more than 90% of SCI cases are traumatic and caused by incidences such as traffic accidents, violence, sports or falls [1–3]. Adults older than 60 years of age whom suffer SCI have considerably worse outcomes than younger patients, and their injuries usually result from falls and age-related bony changes [3, 4]. In this report, a young and healthy male presenting with a penetrating stab injury to his thoracic spine and the management outcome is described with an emphasis on the surgical technique.

## Case presentation

**Fig. 1:**
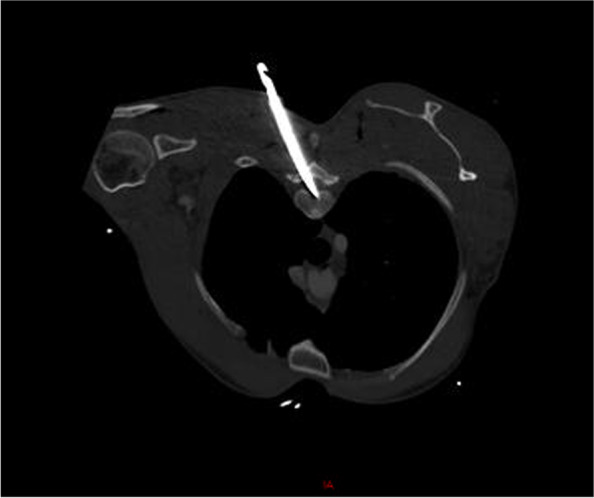
Axial CT scan of the thorax demonstrating foreign body(knife) in the spinal canal in an oblique position reaching the anterior aspect of the spinal canal

**Fig. 2:**
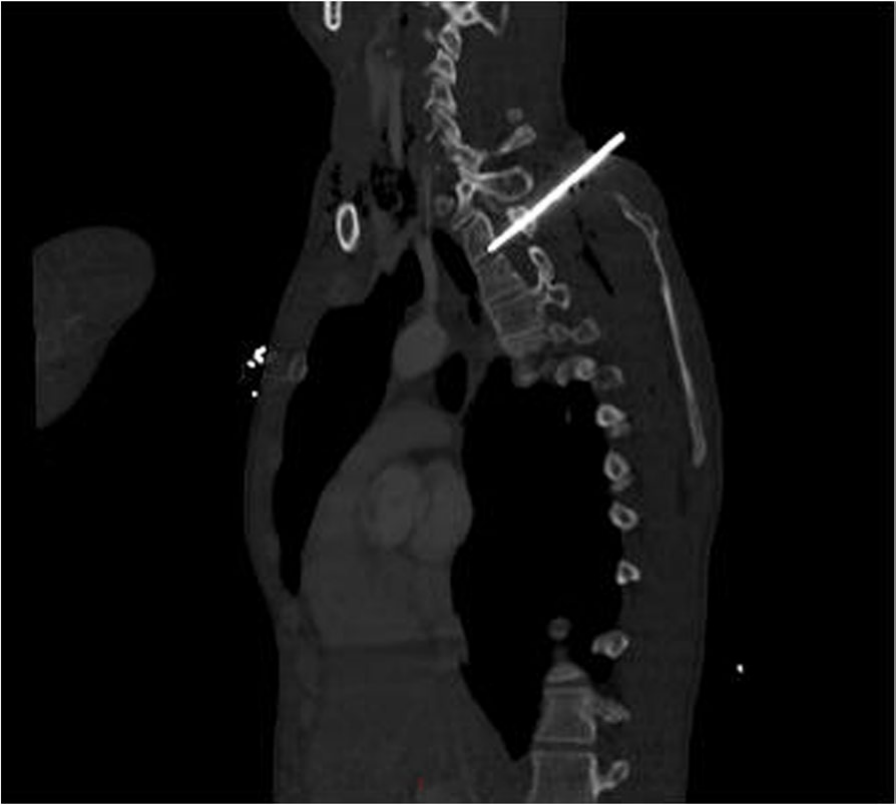
Sagittal CT of thorax demonstrating position of the knife corresponding the T2 and T3 spinal level

A fit and well 34-year-old man was admitted to the emergency service with multiple stab injuries to posterior thorax and occiput with a retained knife in his interscapular area. He remained haemodynamically stable and neurological examination revealed normal power in upper limbs and a 4/5 power in his right lower limb, 0/5 power in his left lower limb. Sensory examination revealed an intact pin prick and light touch from C2-T4, altered sensation T5, absent sensation to pin prick and light touch from T6-S2 with intact perianal sensation. He had a Motor incomplete- ASIA impairment score (AIS) D score. A CT spine was confirmed trajectory of the knife traversing the superior T2 right lamina into left inferior posterior T2 vertebral body. There was no associated vascular injury associated (Figs. 1, 2) .

### Surgical technique

**Fig. 3:**
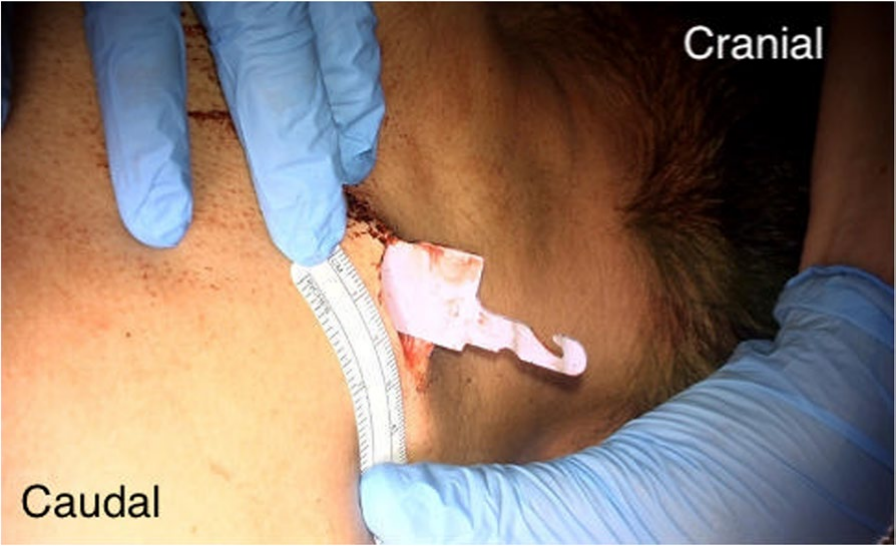
Pre-operative picture of the knife in the interscapular region

**Fig. 4:**
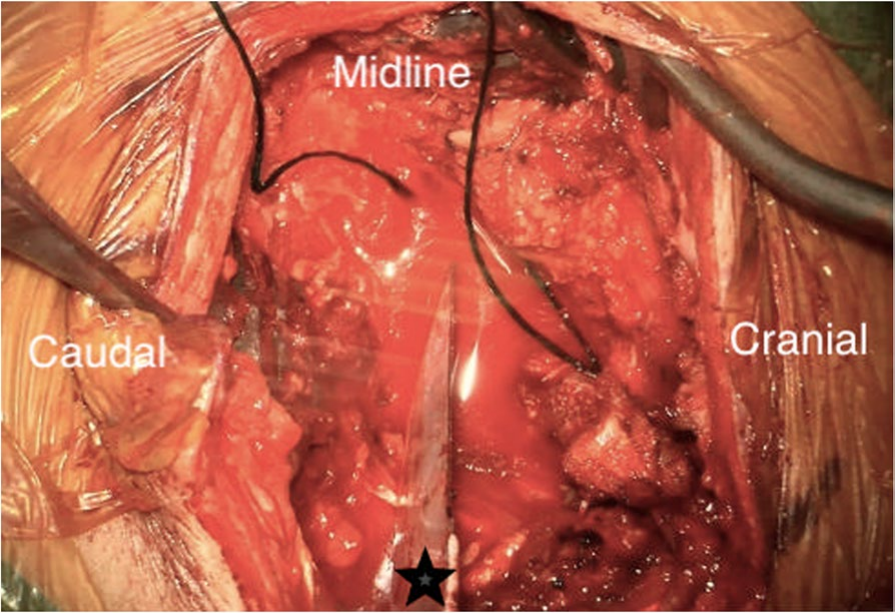
Intra-operative picture after muscle dissection before the laminectomy. Star represents the knife

A thoracic T2 and T3 laminectomy and removal of foreign body was performed within 3 hours of presentation. The knife was noted to be retained in the right paraspinal region with a trajectory of the knife tip to the midline (Figs. 3, 4). A 10 cms midline incision made and connecting the oblique right paraspinal wound. The paraspinal muscles dissected and laminae of T2/3 expose and T2/3 laminectomy completed with high speed drill. 

### Intra operative findings

**Fig. 5:**
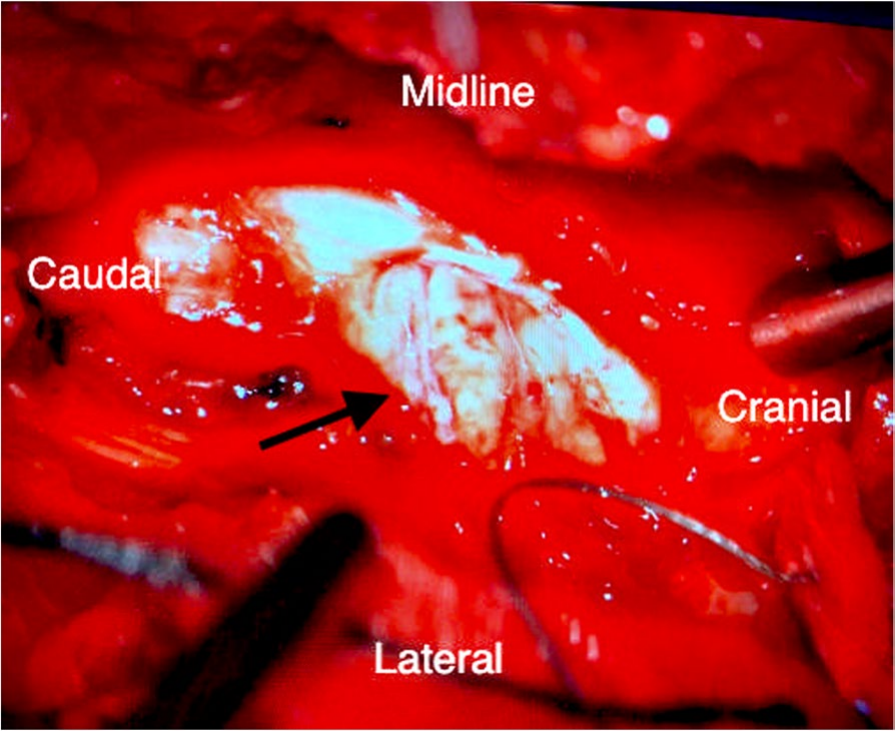
The appearance of the thoracic cord with hemi section on the right side with contused dura. Arrow points to the severed cord. Contused dura is seen in the cranial and caudal portion of the cord

**Fig. 6:**
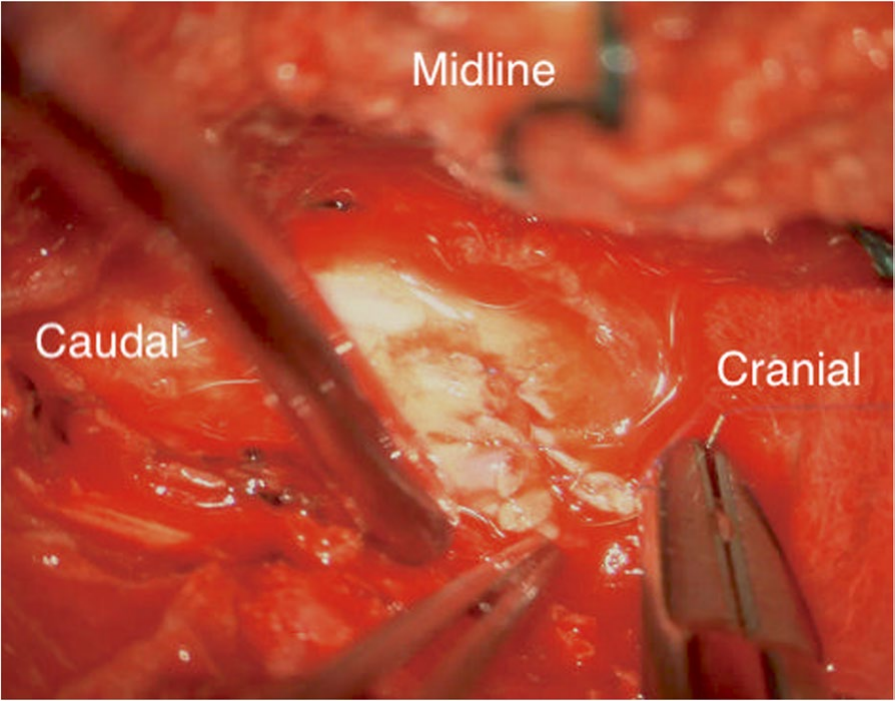
Repair of the cord from medial to lateral with 7′0 prolene

**Fig. 7:**
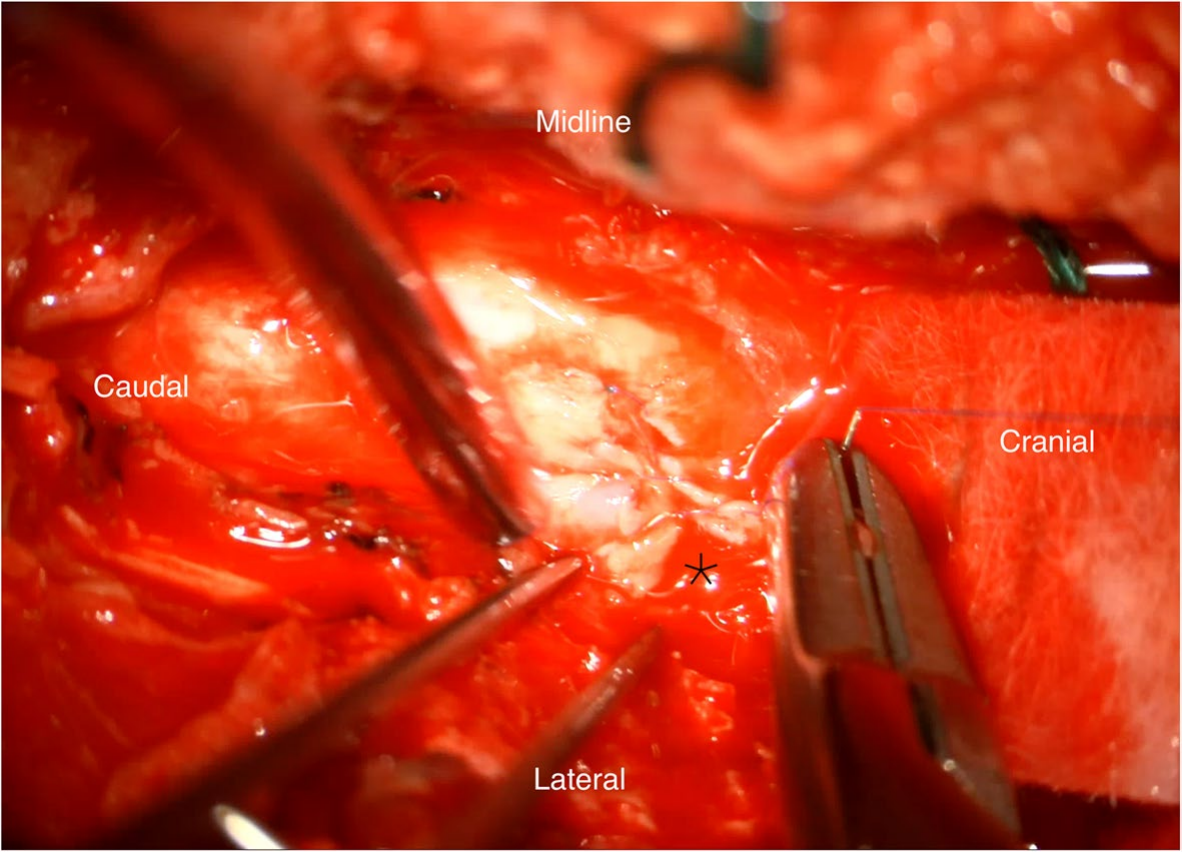
Completion of the repair of the cord. Star represents the lateral portion of the cord with the penultimate suture

Under microscopic guidance, the knife was removed following the laminectomy with minimal manipulatory movement. Care was taken not to move the knife perpendicular to the cord to avoid further damage. The tip of the knife penetrated the right side of the cord causing more than a hemisection (60% of the lateral cord diameter and 100% of anterior posterior diameter on the right side) of the cord landing in the vertebral body and penetrating the posterior longitudinal ligament. The dura appeared contused and severed (Fig. 5). 8 mgs dexamethasone was administered intravenously after the knife was removed from the cord. The torn right hemi cord was sutured with continuous 7′0 prolene (Fig. 6, 7).The pia arachnoidal layer was included in the suturing procedure in a circumferential manner to oppose the torn cord. Haemostasis secured with Floseal®. Dural margins were trimmed and a watertight duroplasty was performed Duraguard™ with 3′0 prolene. Tisseel fibrin glue was used to seal the duroplasty margins. Wound closed in layers. No post-operative surgical complications were encountered.

As this procedure was carried out as an emergency out of hours, neurophysiological monitoring was not utilised.

### Post-operative course

**Fig. 8:**
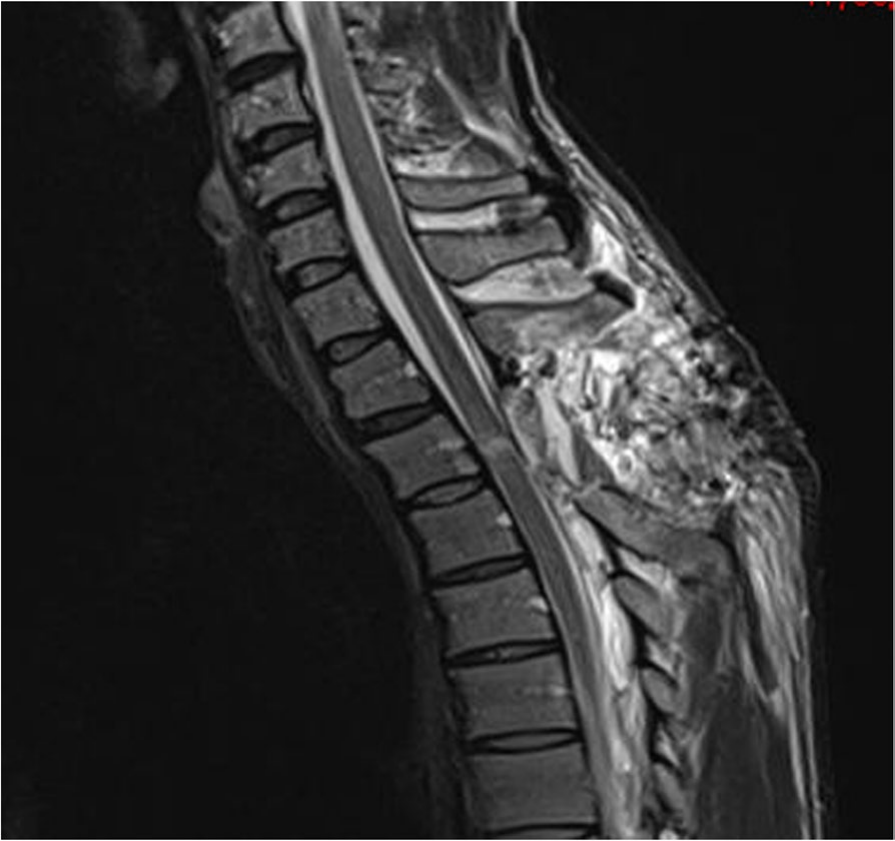
Immediate Post-operative MRI T2 weighted lateral view demonstrating the high signal in the repaired cord and soft tissue changes

**Fig. 9:**
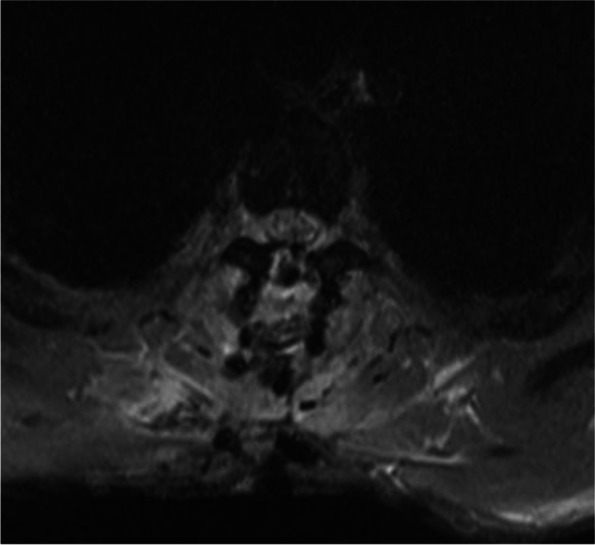
Immediate Post-Operative MRI Axial T2 weighted MRI at T2/3 level demonstrating the cord high signal in the repaired area

**Fig. 10:**
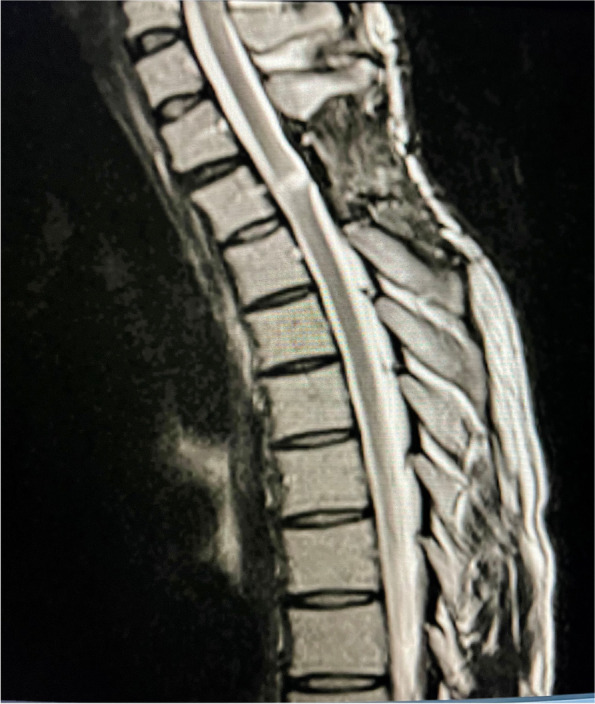
Three month Post-operative MRI T2 weighted lateral view demonstrating the high signal in the repaired cord and soft tissue changes

**Fig. 11:**
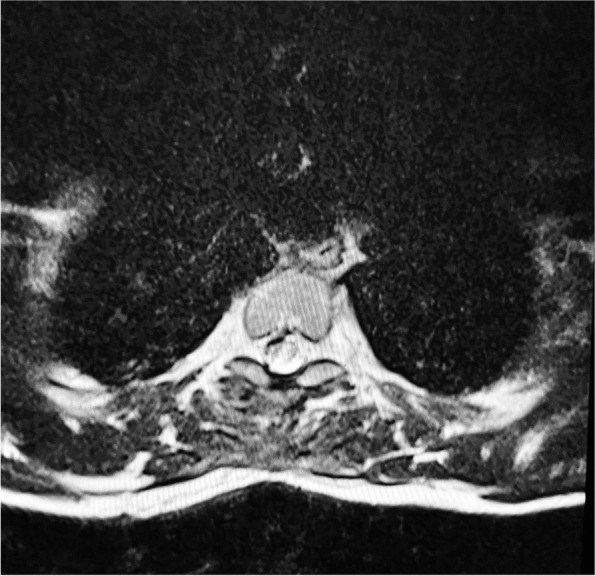
Three month Post-Operative MRI Axial T2 weighted MRI at T2/3 level demonstrating the cord high signal in the repaired area with evidence of healing

He was treated in the intensive care unit and was noted to have complete loss of power in both legs for 8 days and a power of 1/5 noted in his left extensor hallucis longus that improved to 4/5 over 6 weeks. Sensory examination revealed a Brown-Séquard syndrome pattern with loss of pinprick sensation and temperature below T4 on the right side which recovered on the left side. Post-operative spinal MRI at day 4 revealed high signal at the repair sight at the T2/3 level (Figs. 8, 9). An MRI performed at 3 months (Figs. 10, 11) revealed sign of significant spinal cord injury in the and micro-haemorrhages in the cord above and below the level of injury with associated post-operative changes. His ASIA score improved to E from D.

At a 18 month follow-up his is mobilising independently with normal lower limb power and does not need any mobility aid. He is under the care of neuropsychologists for post-traumatic stress disorder (PTSD).

## Discussion

Stab wounds of the spinal cord represent approximately 26% of all spinal cord injuries [1, 5] and remains the most common cause of traumatic Brown-Séquard syndrome [2, 4, 6–10]. In this case, the patient had a picture of post traumatic Brown-Séquard syndrome like picture with complete recovery of the motor functions in a 3 month period with persistent sensory on the right side. It is interesting to note that the patient had 0/5 power in his left lower limb and 4/5 power in his right lower limb likely due damage to ipsilateral and contralateral spinothalamic tracts involvement unlike what is noticed in a pure hemi section of the cord.

Middleton et al. describe in their study that approximately 42% of patients with traumatic SCI have complete dysfunction without any movement or sensation below the site of injury and this may not be applicable to penetrating trauma to spinal cord, nevertheless it gives us an account of the severity of the pathology and clinical implications [11]. They have also eluded to the fact that only 14.3% of all SCIs are believed to be anatomically complete injuries, while the remainder of SCIs are considered as an incomplete functional deficiency with a few spared connections that could be established under proper interventions [11]. In this case, the knife injury had resulted in a hemi section of the cord contributing to incomplete functional status. It is useful to note that the therapeutic options for traumatic SCI includes surgical decompression, anti-inflammatory drugs, hyperbaric oxygen therapy, and rehabilitation interventions [12]. SCI is still associated with a high disability rate despite the intensive rehabilitation programs carried out in hospitals worldwide [3, 12, 13].

In the case described one may argue if microsurgical repair is superior to just removal of the knife without repair of the cord or instillation of fibrin glue to the severed margins. There is no evidence to prove the efficacy of a specific technique leading to good functional recovery. Administration of Dexamethasone in this case is following the observation of the severed cord and the rationale can be questioned. Dural reconstruction following cord repair is vital in preventing post-operative complications compromising recovery.

The clinical outcomes of SCI depend on the severity and location of the lesion and may include partial or complete loss of sensory and/or motor function below the level of injury. Literature describe that cervical level of the spinal cord (50%) with the single most common level affected being C5 and the thoracic level (35%) and lumbar region (11%) in case of Traumatic SCI [1, 11]. In case of penetrating cord injury the aetiology is varied and there is report of penetrating missile injury and management by Kumar et al. [14] where the emphasis on conservative management to surgical removal of the foreign body to avoid iatrogenic deficits. There are reports of accidental penetrating injury to cord secondary to Nail gun injury and wooden fragment penetration causing cauda equina syndrome where role of surgery is described [15–17].

With recent advancements in medical procedures and patient care, SCI patients often survive these traumatic injuries and live for decades after the initial injury [3]. Studies have shown that 40-year survival rate of these individuals was 47% and 62% for persons with tetraplegia and paraplegia, respectively [3, 7]. The life expectancy of SCI patients highly depends on the level of injury and preserved functions. Mary Joan Roach et al. in their recent study have concluded that the patients with penetrating SCI showed more complete injuries and lower surgery rates with worse functional outcome at 1 year [18]. In our case, these evidence gives us an understanding of long term implications in the management of SCI. Kevin Morrow et al. have concluded in their analysis pertaining to penetrating SCI that younger patients are affected and they utilise more health care resources. Surgery is undertaken to limiting progression of neurological deficits, stabilisation and to control infection [19].

In this case, the patient had signs and symptoms of Incomplete cord injury/ Brown-Sequard syndrome that was successfully managed with timely surgical intervention, intense post-operative care and physiotherapy. Neurophysiological monitoring is recommended as an imperative standard adjunct for intramedullary spinal surgery and in this case it was not used as the procedure was carried out of hours as an emergency. One may argue that this may not helpful as there was more than a hemi section in this case and it is likely that the SSEPs, MEPs and D waves would have been significantly compromised or absent.

There is evidence to support that Neuroplasticity plays an important in SCI recovery and physiologically based approach for the rehabilitation of walking has developed, translating evidence for activity-dependent neuroplasticity and the neurobiological control of walking [20, 21]. Neuroplasticity occurs at multiple levels following SCI: Cortical, subcortical, brainstem and spinal cord both short-term and long-term, supporting the need for long term rehabilitation in these cases [21].

There is a paucity of reports eluding to repair of the spinal cord secondary to stab injury and particularly to the thoracic spine. Spinal cord repair is technically feasible however several factors should be considered, particularly the nature of injury, type of the foreign body, age of patient, time to repair from trauma and neurological status of the patient along clear understanding of treatment options. This case demonstrates that with multidisciplinary input, a combination of prompt surgical intervention and rehabilitation has helped an adult patient with penetrating stab injury to the spinal cord.

### Spinal rehabilitation post repair of the cord

Intensive rehabilitation remains the key in recovery of traumatic SCI patients. This is provided a tertiary care centre by a team of dedicated Physicians, Physiotherapists, Occupational therapists, Orthotists, Neurophysiologists and Neuropsychologists. In this case, after the initial period of Intensive post operative care, patient was transferred to the National Spinal Injuries Unit where the majority of recovery took place (https://www.spinalunit.scot.nhs.uk/, https://www.gla.ac.uk/research/az/scisci/). This involved an Intensive inter-disciplinary comprehensive care and patient tailored rehabilitation programme to facilitate the patients’ return to their own community. Once the patient was discharged from the inpatient care, out-patient clinics provided general review, urology, fertility, orthopaedics, neurosurgery, skin, and antispam measures for patients with spasticity. This demonstrates the need for both short and long term rehabilitation input that is essential and it has to be delivered by an experienced multidisciplinary team.

There is recent emerging evidence to prove that non-invasive brain stimulation, as well as spinal cord stimulation, are promising techniques for the rehabilitation of patients with spinal cord injury due to their novelty, effectiveness and minimal side effects [22–24]. In this case, there was no necessity for any stimulation of brain or spine as he showed a remarkable improvement with combination of therapies. He does have spasticity after a 18 month period that is affecting the left limb function to some extent that is currently being managed with low dose of oral diazepam.

## Conclusion

This is the first documented case of hemi-section of the thoracic cord secondary to penetrating knife injury that was successfully repaired and the patient has made a successful functional recovery. The aim of the treatment is to safely remove the foreign body followed by a meticulous and prompt microsurgical repair with water tight duroplasty. The clinical outcome depends on multiple factors including the nature of injury, appropriate preoperative imaging to determine the type of spinal cord injury, clinical judgement and expertise with a suitable post-operative multi-disciplinary treatment facility providing an intensive care and rehabilitation support. The role of spinal neuroplasticity following repair plays a significant role in spinal cord healing and this is supported by provision of high quality intensive medical care. This concurs with recent evidence that in penetrating SCI, surgery is undertaken to limit progression of neurological deficits, stabilisation or infection control. Long term follow-up by the neurorehabilitation team including neuropsychological support is recommended.

## Data Availability

Data sharing not applicable to this article as no datasets were generated or analysed during the current study.
